# Protective Role of Menthol Against Doxorubicin-Induced Cardiac Injury Through Suppression of TLR4/MAPK/NF-κB Signaling and Oxidative Stress

**DOI:** 10.3390/ph19010059

**Published:** 2025-12-27

**Authors:** Mona Mansour, Ahmed M. Ashour, Amany M. Gad, Ali Khames, Shaimaa G. Ibrahim, Mohamed H. A. Gadelmawla, Enas S. Gad

**Affiliations:** 1 Department of Pharmacology and Toxicology, Faculty of Pharmacy (Girls), Al-Azhar University, Cairo 11884, Egypt; 2 Department of Pharmacology and Toxicology, College of Pharmacy, Umm Al-Qura University, Makkah 21955, Saudi Arabia; 3 Department of Pharmacology and Toxicology, Faculty of Pharmacy, Sinai University, Kantara Branch, Ismailia 41636, Egypt; 4 Department of Pharmacology, Egyptian Drug Authority (EDA)—Formerly NODCAR, Giza 12654, Egypt; 5 Department of Pharmacology and Toxicology, Faculty of Pharmacy, Sohag University, Sohag 82511, Egypt; 6 Department of Pharmacology and Toxicology, Faculty of Pharmacy, October 6 University, Giza 12585, Egypt; 7 Department of Life Sciences, Faculty of Biotechnology, Sinai University, Kantara Branch, Ismailia 41636, Egypt; 8 Department of Pharmaceutical Sciences, College of Clinical Pharmacy, King Feisal University, Al-Ahsaa 13889, Saudi Arabia

**Keywords:** menthol, doxorubicin, cardiotoxicity, oxidative stress, inflammation, TLR4, PPAR-α, Caspase-3

## Abstract

**Background/Objectives**: Doxorubicin (DOX) is a highly effective chemotherapeutic agent whose clinical use is limited by dose-dependent cardiotoxicity. This study aimed to investigate the potential protective effects of menthol against doxorubicin-induced cardiotoxicity (DIC) in a rat model. **Methods**: Forty rats were arbitrarily allocated into four groups: (1) normal control, (2) DOX-treated, (3) DOX + menthol treatment, and (4) menthol-only treatment. DOX (15 mg/kg) was applied intraperitoneally, and menthol (100 mg/kg) was applied orally for 7 days following the DOX injection. Cardiac tissue specimens and sera were collected for biochemical assays, histopathological analysis, and immunohistochemistry. Biomarkers of oxidative stress (MDA, GSH), inflammatory pathways (TLR4, MAPK, NF-κB, SREBP-1C), and apoptotic markers (P53, caspase-3) were assessed. **Results**: DOX employment caused remarkable rise in serum troponin levels (6.53 ± 0.98, *p* < 0.05), oxidative stress markers, and inflammatory proteins, alongside histopathological damage in cardiac tissues. Menthol treatment significantly suppressed oxidative stress (MDA, GSH), inflammation (TLR4, MAPK, NF-κB, SREBP-1C levels), and attenuated apoptosis (P53 and caspase-3 expression) (*p* < 0.05). **Conclusions**: Menthol may serve as a promising adjunctive therapy to reduce DOX cardiotoxicity without compromising DOX’s anticancer efficacy.

## 1. Introduction

Doxorubicin (DOX), a potent chemotherapeutic compound, has revolutionized cancer treatment by demonstrating impact in managing a vast range of cancers [1]. Despite its medical benefits, the clinical value of DOX is significantly limited by its dose-dependent cardiotoxic impacts, which cause irreversible injury to cardiomyocytes, ultimately culminating in congestive heart failure [2]. This phenomenon, known as DOX-induced cardiotoxicity (DIC), is a critical and significant concern in clinical oncology. Despite its effectiveness in combating various cancers, DOX’s propensity to cause cardiac damage necessitates a delicate balance between its antitumor efficacy and potential cardiac toxicity [3]. The pathophysiological mechanisms underlying DIC are multifactorial and complex, involving oxidative stress (OS), mitochondrial malfunction, inflammation, and apoptosis in cardiac cells. DOX provokes the production of reactive oxygen species (ROS) which is driven by its iron-dependent mechanism, leading to OS and lipid peroxidation, which disrupt cellular homeostasis and induce cardiomyocyte injury [4,5,6]. Furthermore, DOX interferes with mitochondrial function by inhibiting electron transport chain enzymes, impairing ATP production, and promoting cardiomyocyte apoptosis. Additionally, DOX-induced DNA damage activates stress-responsive pathways, triggering inflammatory responses and contributing to cardiac dysfunction [7,8]. Several preventive strategies and cardioprotective interventions have been studied to mitigate DIC, including dose optimization and concomitant administration of cardioprotective drugs [9,10,11].

The economic and clinical burden of managing DIC underscores the urgent need for effective protective strategies that do not compromise chemotherapeutic efficacy [12].

Furthermore, recent evidence highlights the role of metabolic dysregulation in DIC. Sterol regulatory element-binding protein 1c (SREBP-1c), a master transcription factor for lipid synthesis, is upregulated under oxidative and inflammatory stress, promoting lipid accumulation and further oxidative injury in cardiomyocytes [13]. In contrast, peroxisome proliferator-activated receptor-alpha (PPAR-α), a critical regulator of cardiac fatty acid metabolism, exerts potent anti-inflammatory effects. PPAR-α activation can suppress the NF-κB pathway, and its downregulation is a known feature of cardiac dysfunction [14]. The crosstalk between inflammatory signaling (TLR4/MAPK/NF-κB), metabolic transcription factors (SREBP-1c and PPAR-α), and downstream apoptotic effectors (p53 and caspase-3) represents a plausible, yet under-investigated, integrated mechanism in DIC.

Moreover, emerging therapeutic approaches, such as nanoparticle-mediated drug delivery systems and targeted drug delivery to tumor tissues, hold promise in reducing cardiotoxicity while preserving DOX’s antitumor efficacy [15]. Consequently, clinicians must grapple with the challenge of optimizing cancer therapy while reducing the likelihood of DIC, emphasizing the need for ongoing investigation and the implementation of strategies aimed at mitigating cardiotoxicity without sacrificing treatment effectiveness [15,16].

Current strategies to manage DIC, such as dose limitation and the use of dexrazoxane, are only partially effective and are not without their own limitations and side effects [17].

This has propelled the search for novel, well-tolerated, natural compound-derived adjunctive therapies. Menthol, a monoterpene abundant in mint plants, has garnered attention for its demonstrated pharmacological properties, including potent antioxidant and anti-inflammatory activities in various experimental models [18,19,20].

Wang et al. [21] specifically demonstrated that dietary menthol attenuates cardiac remodeling after myocardial infarction. Menthol’s potential to mitigate DIC stems from its antioxidant, anti-inflammatory, and cytoprotective abilities [20]. These findings provide a strong mechanistic premise for its potential utility in DIC, although its specific role and integrated mechanisms in this context remain largely unexplored [22].

Therefore, this study was designed to investigate the potential cardioprotective effects of menthol against DIC in a rat model. We hypothesized that menthol would confer protection by simultaneously modulating oxidative stress, suppressing the TLR4/MAPK/NF-κB inflammatory axis, rectifying the SREBP-1c/PPAR-α imbalance, and inhibiting the subsequent apoptotic cascade. Elucidating this multi-targeted mechanism will provide a comprehensive scientific foundation for considering menthol as a promising candidate for adjunctive therapy in DOX-treated patients.

## 2. Results

### 2.1. Impact of Menthol on Serum Level of Troponin-1 DIC

**Figure 1:**
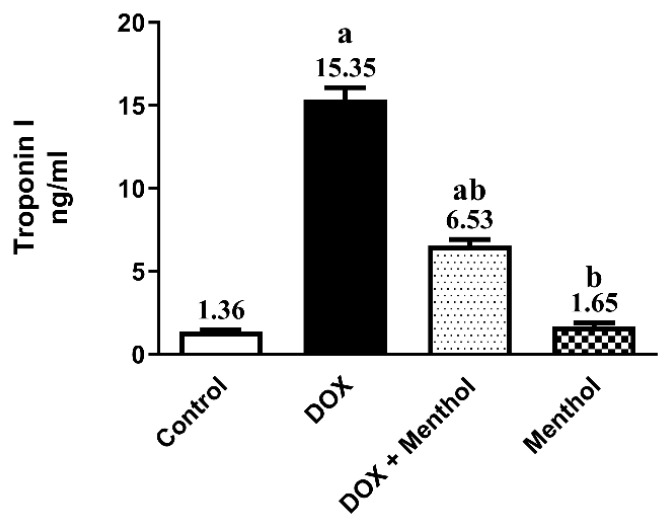
Effect of menthol on serum Troponin-1 (A) level. Data were expressed as mean ± S.E.M (*n* = 10). Menthol was given at a dose (100 mg/kg/p.o./daily) for 7 days. Blood and tissue samples were collected on day 8. Cardiotoxicity was induced by DOX (15 mg/kg, IP, once). Statistical analysis was performed using ANOVA followed by Turkey’s multiple comparison test. (*n* = 10) a: substantially varied from the control group, b: substantially varied from DOX group at *p* < 0.05.

Troponin-1 serum levels were significantly elevated in rats administered DOX (15 mg/kg, i.p., once) by 11.3-fold compared to the normal control (15.35 ± 1.78). The group that received menthol (100 mg/kg, p.o., daily for 7 days) showed a remarkable decline in Troponin-1 levels by 57.5% compared to the DOX group (6.53 ± 0.98) (Figure 1).

### 2.2. Impacts of Menthol on OS Markers Towards DIC

**Figure 2:**
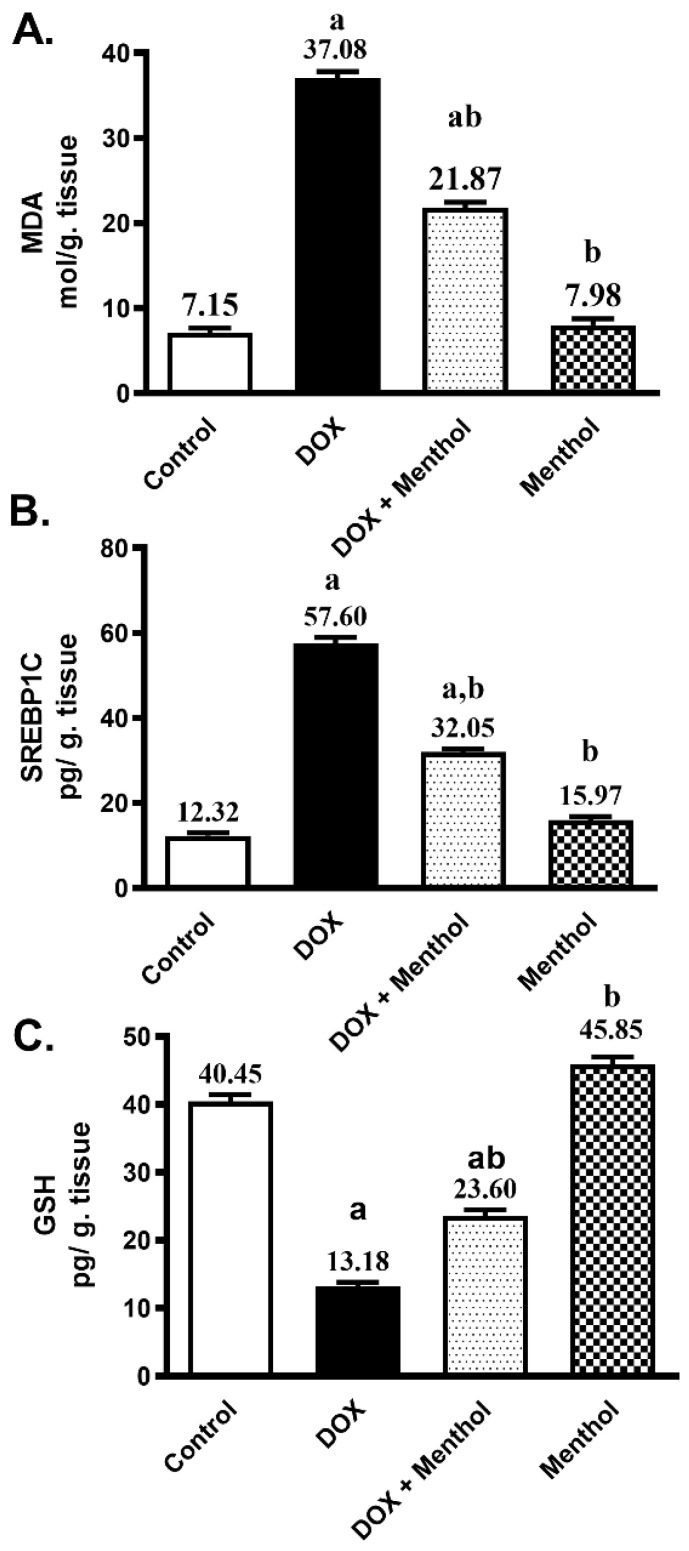
Effect of menthol on MDA (**A**), SREBP1C (**B**), and GSH (**C**) contents. Data were presented as mean ± S.E.M (*n* = 10). Menthol was given at a dose (100 mg/kg/p.o./daily) for 7 days. Blood and tissue samples were collected on day 8. DIC (15 mg/kg, IP, once). Statistical analysis was performed using ANOVA followed by Turkey’s multiple comparison test. a: substantially varied from the control group, b: substantially varied from DOX group at *p* < 0.05.

The antioxidant potential of menthol against ROS production provoked by DOX was evaluated by assessing the contents of MDA, SREBP1C, and GSH. DOX administration produced a remarkable rise in both cardiac MDA and SREBP1C contents by approximately 5.2-fold and 4.7-fold, respectively, as well as a significant decrease in GSH by 67.4% compared to the control. The DOX group showed elevated MDA (37.08 ± 1.81) and SREBP1C (57.60 ± 3.38) levels, along with a marked reduction in GSH (13.18 ± 1.50) (Figure 2). Menthol administration markedly reduced the cardiac MDA and SREBP1C levels by 41% and 44.4%, respectively (21.87 ± 1.47 and 32.05 ± 1.83), and prevented the reduction in GSH content by about 79% (23.60 ± 2.11) compared to the DOX group (*p* < 0.05).

### 2.3. Impacts of Menthol on Inflammatory Markers Towards DIC

**Figure 3:**
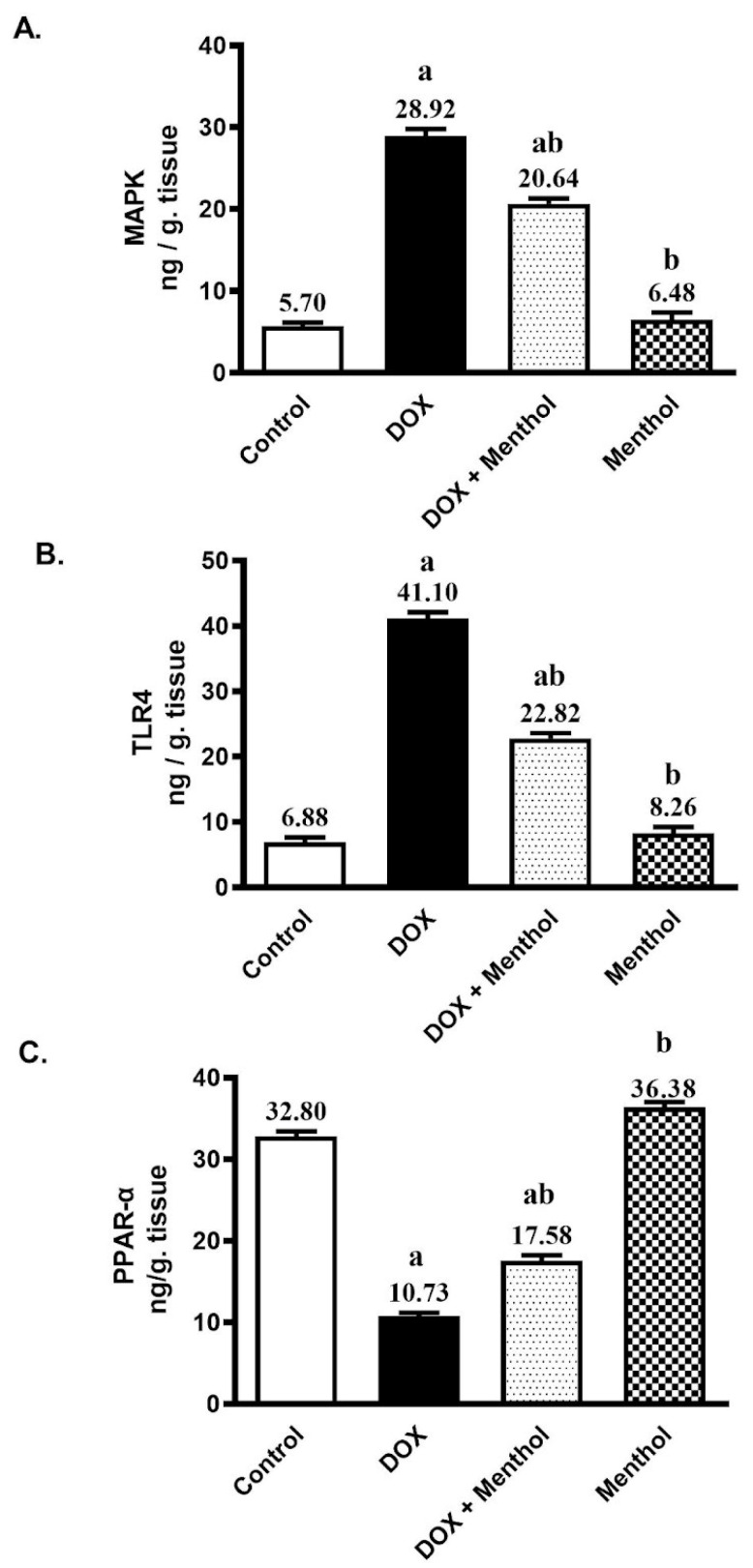
Effect of menthol on MAPK (**A**), TLR4 (**B**), and PPAR-α (**C**) contents. Data were expressed as mean ± S.E.M (*n* = 10). Menthol was given at a dose (100 mg/kg/p.o./daily) for 7 days. Blood and tissue samples were collected on day 8. Statistical analysis was performed using ANOVA followed by Turkey’s multiple comparison test. a: substantially varied from the control group, b: substantially varied from DOX group at *p* < 0.05.

DOX substantially increased the cardiac contents of MAPK and TLR4 by about 5.1-fold and 6-fold respectively, as well as significantly reduced cardiac PPAR-α by 67.3% compared to the control group. The DOX group showed elevated MAPK (28.92 ± 1.96) and TLR4 (41.10 ± 2.25) levels, along with a marked reduction in PPAR-α (10.73 ± 1.10) (Figure 3). Menthol administration revealed a substantial hampering in the cardiac contents of MAPK and TLR4 by 28.6% and 44.5%, respectively (20.64 ± 1.52 and 22.82 ± 1.79), and prevented the reduction in PPAR-α content by about 63.8% (17.58 ± 1.54) compared to the DOX group (*p* < 0.05).

### 2.4. Evaluation of Immunohistochemistry Changes

**Figure 4:**
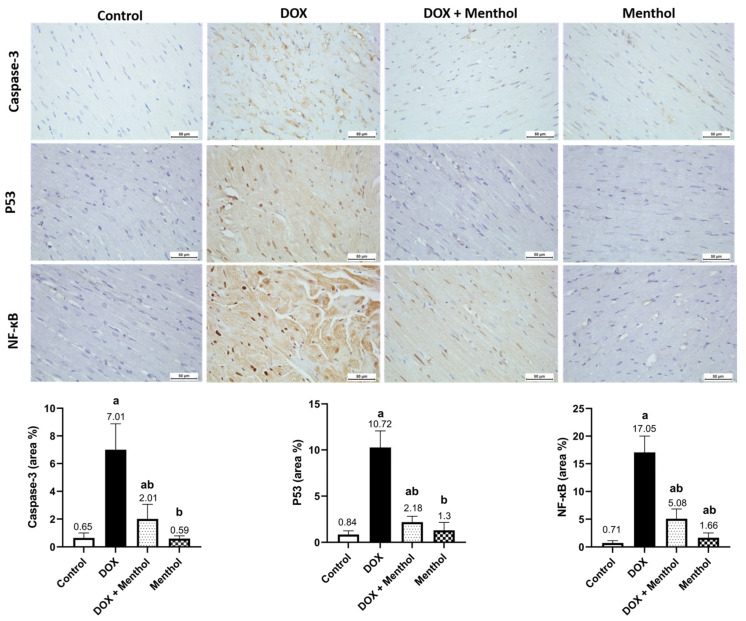
Photomicrographs of heart tissues showed weak Caspase-3, p53, and NF-κB expression in control and menthol groups. The DOX group exhibited severe p53 but moderate Caspase-3 and NF-κB expression, while the DOX + menthol group revealed weak, near-normal expression (DAB, ×400). Values are mean ± SEM at *p* <0.05. a: substantially varied against control, b: substantially varied against DOX group.

IHC examination revealed weak Caspase-3, p53, and NF-κB reactivity in the control and menthol groups (Figure 4). The DOX group showed severe expressions of p53 and NF-κB and moderate Caspase-3 reactivity, with measured levels of p53 (10.27 ± 1.80), Caspase-3 (7.01 ± 1.88), and NF-κB (17.05 ± 2.96). The treatment group (DOX + menthol) exhibited weak Caspase-3 and p53 expression and mild NF-κB reactivity, with corresponding levels of p53 (2.18 ± 0.64), Caspase-3 (2.01 ± 1.05), and NF-κB (5.08 ± 1.77), as compared with the DOX group. There was a notable variation between the DOX and treatment groups concerning Caspase-3, p53, and NF-κB expression (*p* < 0.05), reflecting a substantial decrease in positivity for all three markers in the treatment group.

### 2.5. Evaluation of Histological Changes

**Figure 5:**
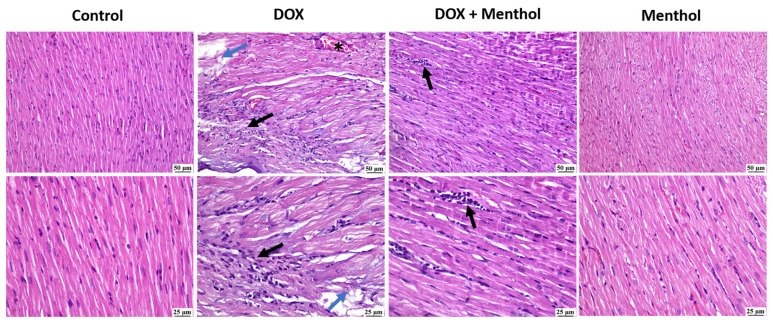
The photomicrographs show the cardiac tissue from various groups of experiments. The control group shows normal myocardium. In contrast, the DOC group reveals myocardial necrosis with fragmentation (blue arrows), infiltration of mononuclear cells (black arrow) at both magnifications, and congested blood vessels (*). The DOX + menthol group showed small focal aggregations of mononuclear inflammatory cells (arrows) at both magnifications and apparently normal myocardium. The menthol-alone group exhibits clearly normal myocardium. (H&E, ×400).

No histopathological alterations were detected in the assessed heart samples of the control group (Figure 5). Similarly, an apparently normal myocardium was observed in the menthol-only group. The DOX group revealed intense mononuclear inflammatory cells infiltration, myocardial degeneration, and necrosis. Degenerating muscles appeared hyalinized, fragmented with pyknotic nuclei. The DOX + menthol group was improved as the examined myocardial sections revealed congestion and small focal aggregates of mononuclear inflammatory cells in a few sections with apparently normal myocardium in most of the examined.

## 3. Discussion

DOX is one of the highly efficient antitumor agents that can kill cancer cells for many types of cancer, including hepatic and breast cancer, and DOX is very effective in the treatment of hematological cancers [23,24,25]. This demonstrates its importance and our inability to practice without it, but, unfortunately, its serious induced cardiotoxicity restricts its use. The patient is harmed by the DIC more than by the cancer itself [26,27]. DOX administration induced cardiotoxicity in rats as evidenced by increased serum levels of troponin-1, and, as demonstrated by Baniahmad et al. [28] and Linders et al. [29], this can be attributed to the basis of leakage of troponin and other cardiac enzymes from cardiomyocytes after its damage. Oxidative stress is the main mechanism behind DIC due to the production of free radicals from DOX metabolites; this is proved in our study by the increase of OS marker MDA and a decline in GSH in cardiac tissues. This concurs with Khames et al. [30], and the phenomenon can be attributed to the production of semiquinone metabolite of DOX that reduces the molecular oxygen and leads to superoxide (O2--) production, which togetherstart a great cascade of oxidative reactions generating ROS that finally send cardiac cells to inflammation or apoptosis step [4,31]. In our study we evaluated DOX cardiotoxicity in the context of the integrated and the consequent on each other pathway: TLR4/MAPK/ NFKB and SREBP.1C/PPAR. It is worth noting that no other researchers addressed such an interconnection mechanism. TLR4 is a transmembrane protein that is stimulated by immune stimuli and foreign bodies; after stimulation it ignites a critical inflammatory pathway starting with MAPK P38 that is a protein kinase phosphorylates and activates the transcriptional factor NF-κB which codes for inflammatory mediators production and induces cardiomyocytes inflammation and remodeling [32,33,34]. SREBP.1C is a transcriptional factor that codes for the production of sterols and lipids in the liver [35,36]. It is also associated with increased oxidative stress through increased lipid peroxidation [37,38,39], NF-κB-activated SREBP.1C in the diabetic nephropathy model [40], and SREBP.1C-boosted NF-κB in a model of colorectal cancer [41]. PPAR-α inhibits the generation of several pro-inflammatory cytokines and mediators; it inhibits NF-κB [42]. PPAR-γ has been established as having a critically crucial function in the immune response by inhibiting TLR4 activation and inflammatory cytokines release [43]. It also has been established to suppress SREBP.1C activation because PPAR –α and SREBP.1C are physiologically inversely related to each other—PPAR –α decreases and optimizes blood glucose and lipids while SREBP.1C elevates them.

DOX caused elevated values of TLR4, MAPK, NFKB, and SREBP.1C in agreement with Rahmatollahi et al. [44], Shi et al. [31], Arab et al. [45], and Wang et al. [46]. This can be explained based on the inflammation induced by DIC where OS caused by DOX semiquinone metabolites exacerbate the inflammatory response in cardiac tissues and upregulate inflammatory markers. DOX decreased the cardiac tissue levels of PPAR–α as with Renu et al. [47] and Wang et al. [46]. This can be understood due to the anti-inflammatory role of PPAR –α activation and how it leads to the suppression of TNF-α, IL-6, and IL-1β by reducing the activation of NF-kB and AP-1 activation, which are essential in inflammatory pathways [48,49,50].

DOX employment caused elevated values of P53 and caspase-3, and this is in accordance with Yun et al. [51] and Yang et al. [52]. P53 is a tumor suppressor protein and an apoptotic inducer; stimulation of cellular injury by doxorubicin results in DNA damage and so activates P53 which in turn activates caspase-3. P53 can activate Caspase-3 by another mechanism through the transcription of certain genes involved in apoptosis, in addition to abnormalities of cardiac tissues markers. A histopathological study proved the cardiotoxic effect of DOX where the DOX group showed intense mononuclear inflammatory cells infiltration, myocardial degeneration, and necrosis. Degenerating muscles appeared hyalinized, fragmented with pyknotic nuclei. While the DOX + menthol group was improved as the examined myocardial sections revealed congestion and small focal aggregates of mononuclear inflammatory cells in few sections with apparently normal myocardium in most of the examined individuals.

**Figure 6:**
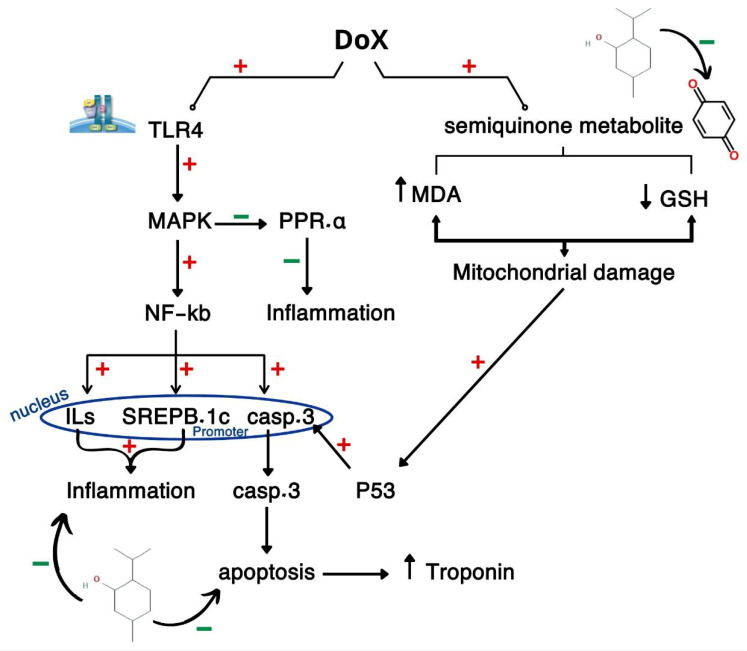
Graphical abstract illustrates the pathway for DIC through crosstalk between CAMP, PI3K/AKT, and FOXO.1, PTP.1B /p53/caspase-3.

Menthol ameliorated DOX-cardiotoxicity through decreasing serum levels of troponin-1 in agreement with Osman et al. [53] and Wang et al. [21]. This can be proved on the basis of the cardioprotective and antihypertrophic effects of menthol [54]. Menthol normalized the cardiac levels of MDA and GSH, and this is in line with Khames et al. [55], Almatroodi et al. [56], and Wulandari et al. [57]. This is attributed to the antioxidant efficacy of menthol, its free radical scavenging ability, and lipid peroxidation inhibition effect. Menthol administration decreased the inflammatory response that is induced by DOX through decreasing cardiac tissue levels of TLR4, MAPK, NFKB, and SREBP.1C as discussed by Goudarzi et al. [58], Reale et al. [59], and Takasawa et al. [60]. These results can be attributed to menthol’s anti-inflammatory effect where it is proved to decrease TNF-α and IL-1β [18,61]. Menthol increased significantly the levels of PPAR-α, and this confirms the anti-inflammatory activity of menthol. Menthol administration attenuated the apoptotic response induced by DOX through decreasing the oxidative potential of DOX and its metabolites, preventing cardiac cell DNA damage through inhibiting both P53 and caspase-3 as described by Rozza et al. [61] and Matouk et al. [20]. DOX provoked OS which initiates the activation of the TLR4, which, upon activation, initiates a cascade of signaling events, including the MAPK pathway which, in turn, leads to the translocation and NF-κB activation that induces pro-inflammatory cytokines’ expression and contributes to inflammation and cell survival signaling. Concurrently, SREBP-1c, a regulator of lipid synthesis, is upregulated, which further exacerbates cellular stress by promoting lipid accumulation and dyslipidemia. SREBP-1c is induced by NF-κB, and both provoke inflammatory response in cardiac cells. At the same time, it promotes MAPK phosphorylates and inhibits PPAR-α, exacerbating inflammation. DOX is metabolized into semiquinone form that starts an oxidative cascade through ROS generation, increased lipid peroxidation, and MDA, and consumed GSH contribute to mitochondrial damage, cell cycle arrest, and apoptosis with the activation of p53, which provokes caspase-3 expression and apoptosis of heart tissues due to the leakage of intracellular contents as troponin from damaged tissues. On the contrary, menthol attenuated DIC by reversing OS, inflammation and apoptosis and normalized histopathological pictures as shown in Figure 6.

The present study has certain limitations that should be acknowledged. First, an acute rather than a chronic model of DIC was used, which may not fully represent the cumulative and progressive cardiac effects observed in clinical settings. Second, functional cardiac assessments, such as echocardiography or electrocardiographic evaluation, were not performed; therefore, the current findings reflect biochemical and histological improvements but cannot confirm functional recovery. Third, pharmacokinetic and pharmacodynamic data for menthol were not evaluated, limiting conclusions regarding its systemic distribution and dose–response relationship. Future studies should therefore focus on employing chronic experimental models, integrating comprehensive cardiac function analyses and elucidating menthol’s pharmacokinetic profile to validate and extend its potential as a cardioprotective candidate. The findings of this study provide mechanistic insights into the cardioprotective potential of menthol against DOX-induced toxicity. By modulating oxidative, inflammatory, and apoptotic signaling cascades, menthol demonstrated significant biochemical and histopathological improvements. Although these outcomes are promising, they represent preclinical proof of concept rather than clinical evidence. Translating these findings to human applications will require comprehensive evaluation of menthol’s pharmacokinetics, optimal dosing, safety, and efficacy through chronic and functionally validated models.

## 4. Materials and Methods

### 4.1. Animals

The study utilized 40 male Wistar rats weighing (200–250 g), supplied by the National Organization for Drug Control and Research (NODCAR) in Cairo, Egypt. Rats were acclimatized in a controlled environment with a 12 h light–dark cycle and had free access to standard chow and water ad libitum. The housing conditions were controlled, with room temperature of 23 ± 2 °C and humidity maintained at 60 ± 10%. All experimental procedures received ethical approval from the Faculty of Pharmacy, Sinai University, with authentication no (SU. REC.2024. 36 A) (13 January 2025) and were performed in accordance with the International Guiding Principles for Biomedical Research Involving Animals (CIOMS & ICLAS), maintaining adherence to the NIH Guide for the Care and Use of Laboratory Animals (8th edition, revised 2011). All animal research approaches were performed in line with the ARRIVE standards and were authorized by the Research Ethics Committee. Every effort was made to minimize any discomfort, pain, or suffering for animals throughout the study.

### 4.2. Drugs and Chemicals

Doxorubicin hydrochloride (CAS 25316-40-9) and menthol (CAS 89-78-1) were commercially sourced from Sigma-Aldrich Chemical Co. (St. Louis, MO, USA). All other chemicals used were of analytical grade and high purity.

### 4.3. Experimental Design

Forty rats were randomly allocated into four experimental groups (n = 10 per group). Group I (normal control) received the vehicle (1% carboxymethyl cellulose, CMC) orally for 7 days and a single i.p. injection of saline (5 mL/kg) on day 1. Groups II (diseased model) was administered a single i.p. dose of DOX (15 mg/kg) to induce cardiomyopathy [62]. Group III served as treatment where DOX-exposed rats received menthol (100 mg/kg/day; orally., once daily) 60 min after DOX injection for 7 consecutive days [63]. Group IV served as drug control and were normal rats given menthol (100 mg/kg/day, P.O., once daily) for 7 days.

### 4.4. Sampling and Tissue Collection

By the completion of the experimental protocol, blood was withdrawn from the retro-orbital plexus of anesthetized rats. After a 20 min clotting period at room temperature, the blood tubes were centrifuged at 503× *g* for a duration of 15 min. The resulting supernatant (serum) was carefully separated and immediately preserved at −80 °C for subsequent biochemical assessment. Then after blood collection, euthanasia was performed on the animals following a deep anesthetic dose (1 mL i.p. of 0.3 mL xylazine and 0.7 mL ketamine). The hearts were rapidly dissected out and divided into two sections: one was immersion-fixed in formalin for histopathological assessment, and the other was snap-frozen at −80 °C for analysis by ELISA. The cardiac tissue was homogenized using a polytron homogenizer, following the mixing of 50 mg of cardiac tissue with cold lysis buffer (NP40 (1%), SDS (1%), Tris-HCl, pH 8.0, sodium deoxycholate (0.5%), NaCl, and phenylmethylsulfonylfluoride) [64].

### 4.5. Quantitative Determination of the Rats’ Serum Levels of Troponin

The rats’ troponin-1 blood levels were measured utilizing rat-specific ELISA kits (Cat#: MBS727624; My BioSource Co., San Diego, CA, USA). The assay was conducted as directed by the manufacturer. Troponin1 was selected as it is a highly specific and sensitive biomarker for myocardial injury

### 4.6. Biochemical Assays in Cardiac Tissues

The levels of MDA and reduced GSH were quantified in cardiac tissues of DOX-exposed rats using the ELISA assay technique. Malondialdehyde ELISA kit was purchased from My BioSource company (My BioSource, San Diego, CA, USA, Cat #: MBS268427); while GSH ELISA kits were acquired from BlueGene Biotech company (BlueGene Biotech Co., Ltd., Shanghai, China, Cat#: E02G0367). The assessment procedures followed the manufacturers’ instructions per each biomarker kit.

The heart tissue concentrations of PPAR-α (Cat #: LS-F9236; LSBIO Co., Seattle, WA, USA) and SREBP-1C (Cat #: MBS940898; My BioSource Co., San Diego, CA, USA) were assessed employing rat-specific ELISA assay kits. The assay was conducted according to the supplied instructions.

Cardiac tissue levels of TLR4 and MAPKs were assessed with the following rat-specific ELISA kits, following the manufacturers’ directions. The kits were obtained from BioSource Company; (Cat #: MBS940898; My BioSource Co., San Diego, CA, USA) for TLR-4 and (Cat #: MBS705488; My BioSource Co., San Diego, CA, USA) for MAPKs.

### 4.7. Immunohistochemistry (IHC)

For immunohistochemical analysis, 5 µm thick heart sections from all experimental groups were incubated with primary antibodies for Caspase-3 (CAT # 700182; ThermoFisher Scientific, Cambridge, MA, USA, 1:50), p53 (CAT # MA5-12453; ThermoFisher Scientific, Cambridge, MA, USA, 1:20), and NF-κB (CAT # MA5-41097; ThermoFisher Scientific, Cambridge, MA, USA, 1:200) for 90 min. This was followed by a secondary antibody applied using the immunoperoxidase method. Staining was completed with a Universal DAB Staining Kit (ScyTek Laboratories, Inc., Logan, UT, USA) for 30 min at room temperature [65,66,67].

### 4.8. Quantitative Evaluation of IHC Staining

IHC Semi-quantitative analysis is an effective tool for studying protein expression and location inside tissues performed on photomicrographs of six non-overlapping fields per specimen employing ImageJ Fiji software version 1.2 (no special plugin was utilized). The area percentage of positive Caspase-3, p53, and NF-κB immunoreactivity was assessed at X 400 magnification for all samples [64]. A blind image analysis was conducted to limit bias.

### 4.9. The Histopathological Examination

For histological assessment, hearts were rapidly dissected out and immersion-fixed in 10% neutral buffered formalin and underwent histopathological examination through staining with hematoxylin and eosin (H&E). This process allows for the visualization of tissue structures and cell morphology [66,68].

### 4.10. Statistical Analysis

All results were analyzed and assessed with Graph Pad Prism Version 5 (San Diego, CA, USA). The results were presented as the mean and standard error (S.E.), and the results were analyzed using one-way ANOVA. Tukey’s post hoc test was used to assess differences across groups. *p*-values < 0.05 were considered significant.

## 5. Conclusions

Menthol exhibits a potential cardioprotective effect against DOX-induced injury by modulating the TLR4/MAPK/NFκB/SREBP-1c/P53/caspase-3 signaling network. It attenuated oxidative stress and inflammation while enhancing PPAR-α expression, which plays a key anti-inflammatory and metabolic regulatory role. Moreover, menthol mitigated apoptosis through the downregulation of P53 and caspase-3, thereby preserving myocardial integrity. Although these findings reveal biologically significant protective actions, the translational and therapeutic implications should be considered hypothetical until validated with chronic models and functional cardiac assessments.

## Data Availability

The original contributions presented in this study are included in the article. Further inquiries can be directed to the corresponding authors.

## Abbreviations

The following abbreviations are used in this manuscript:

DOX	Doxorubicin
DIC	Doxorubicin-induced cardiotoxicity
MDA	Malondialdehyde
GSH	Glutathione
TLR4	Toll-like receptor 4
MAPK	Mitogen-activated protein kinase
NF-κB	Nuclear factor kappa B
ROS	Reactive oxygen species
OS	Oxidative stress
PPAR-α	Peroxisome proliferator-activated receptor alpha
TNF-α	Tumor necrosis factor alpha
IL-6	Interleukin 6
IL-1β	Interleukin 1 beta
ELISA	Enzyme-Linked Immunosorbent Assay
ANOVA	Analysis of variance
P.O	Per Os
i.p.	Intraperitoneal
SREBP-1c	Sterol Regulatory Element-Binding Protein-1c
